# Excision v. Extraction

**Published:** 1885-12

**Authors:** C. Spence Bate


ARTICLE II.

### EXCISION *v.* EXTRACTION.

BY MR. C. SPENCE BATE, F. R. S.

At a time when surgery is conservative in its treatment, and the greatest efforts are being made to retain parts that are only of secondary value, it is remarkable that in dental surgery the instruments available for the forcible removal of a tooth from its natural position have of late years so largely increased.

That many of these are intended to expedite the operation and lessen the pain, is without doubt true, but it also surely evidences a foregone conclusion, that an operation being convenient, is more likely to be determined upon.

It is now some fifty years since a Mr. Fay of Liverpool, introduced into his practice the mode of excision of the teeth, in order to obviate the pain of extraction, but as his mode of treatment was, after the operation, to allow the roots of the teeth to take care of themselves, it was found that he had to remove a large number of the retaining stumps for the purpose of getting rid of abscesses, gum boils, &c.: circumstances that induced him to return to the more common mode of practice, and extract the teeth bodily, rather than allow a portion to remain at the risk of future trouble and a second operation.

I have given this account of Mr. Fay's practice, not from any knowledge of my own, but from what I have heard when I was younger; but it should be taken into consideration that when Mr. Fay experimented on excision, the power of conserving the remaining stumps was not within the bounds of practical surgery, as it has since become.

Long before Mr. Fay attempted the excision of the teeth that are situated in the posterior portion of the jaws, it was carried out and is still continued in relation to the teeth with simple roots, which are largely retained for the purpose of supplying the loss of their crown with an artificial substitute.

If the operation be desirable and capable of being effectively pursued in the anterior portion of the mouth, there is no reason why it cannot be as successfully fulfilled at the posterior, the only reason, as far as I can see, is that our patients generally are in accordance with us in our practice in the former case, but are largely antagonistic in the latter, which coincides with the more convenient and easier mode of preparing the jaws for the reception of substitutes than that which entails a prolonged demand on our time in the necessary treatment that the stumps may require. Another apparently satisfactory reason for the removal of the stumps is the self-satisfying argument that when they are taken out " they are certain not to pain again ; " but there are other effects which an extensive series of extractions will produce that, I think, are of greater permanent evil than the risk of local disturbance that may follow the retention of an unsuccessfully treated stump or series of roots.

The amount of absorption that follows the loss of a tooth by extraction is very considerable, and when a large number are removed at the same time, the waste of osseous tissue is in a much greater ratio than in relation to the same number of teeth when extracted at various times. Nor is this the worst, for sometimes the waste goes on year after year, to certainly a less extent than at first, but so continuously that in some cases the tuberosity for the attachment of the lingual frenum may be seen conspicuously elevated as a prominent spine. Nor is this conspicuous waste of bone the worst possible feature, for in some instances the shock which the system receives is so great that it is sometimes long in its recovery. One such case has been brought to my notice within the last twelve months that terribly elucidates my meaning.

I learned from the patient herself that she had consulted me a year or so previously relative to her mouth, and that I had advised her to have no teeth removed, but some eight or nine substitutes inserted. She was then probably on a tour of professional interviews, and ultimately appears to have fixed on one who would do for her the largest amount of work for the smallest amount of payment. The result was that she had every tooth but two second lower molars extracted, and when I saw her again the alveolar ridge was wasted to a small and narrow line round the jaw, and she was wearing an imperfectly-fitting set of teeth, arising from the large amount of absorption that had gone on since the substitutes had been inserted.

One prevailing idea the patient had was that he who had extracted her teeth had ruined her for life, that he had deformed her in face and body, that she was fast becoming an unsightly person. To quote one of several letters that I received from her, soon after I had placed in her mouth another set. She writes:—

"I do not wish to trouble you with any correspondence, but I think my case needs an apology—I am grievously disappointed in the results. When I told you that I wished to be bigger, I was so pent up in the body that I could not understand for what cause I had been so treated and had so suffered.

"Now I find I am getting very stout every way, and that which had been brought almost to a climax, and which would have been very beneficial to me, has all dispersed, and in order to make me stouter I find my body and face are distorted. My nose is still waving to and fro, which I supposed is acting on my spine and causing it to be knotted and crooked.

"My face is all on one side, as is also my body.

"I am almost afraid to wear my teeth, the vulcanite is expanding so, and I feel that with what is going on in my mouth I am further away from having my desires realized."

The writing of one such letter as the above would be suggestive of an exhibition of feeling arising from some annoyance connected more or less immediately with the distress induced by the novel sensation of wearing an extensive set of substitutes for the first time. But when such letters are repeated and continued for a considerable period, when, added to this, the existence of the patient has become a burden to herself and friends, that medical treatment has had no power to remove the melancholy, that she has been compelled to have an attendant in constant association with her, I think we are forced to attribute this destressing monomania as the consequence of the removal of a large number of teeth and the great immediate personal change which a considerable amount of alveolar absorption induces.

Conservative dentistry is, I believe, the better surgery, and where the roots of teeth are retained in a healthy condition the mouth is preserved in a higher degree of efficiency, both for personal appearance, ease and comfort, as well as for the satisfactory application of artificial substitutes.

Unfortunately it has been too common a practice for the stumps that are left in the mouth, being either the remains of decayed teeth or others that had been excised to admit of their replacement by substitutes, to be allowed to shift for themselves. The natural channel of the dental pulp is allowed to exist either as an open chamber to be occupied with oral deposits, or to retain the slough of the dental pulp and so pave the way to future periostitis and alveolar abscess.

Nor is the dentist wholly responsible for this condition of things, inasmuch as the general anxiety of the patient is not that the mouth shall be placed in a thoroughly healthy condition, but that the greatest amount of show shall be made for the least amount of personal inconvenience. Consequently all things like decayed stumps are allowed to take their chance, and a pus-discharging gumboil is looked upon as a valued safety-valve against active pain.

It is not my intention to raise an indiscriminate determination that no tooth or stump should ever be extracted, but I do think that neither stump nor tooth should be removed that is healthily implanted in its alveolus or could be made so. Roots that are loose, roots that are inducing induration of the periodonteum, roots that are the exciting causes of a chronic inflammatory condition of the gums, or are distantly connected with obscure pains of the head and various parts of the sysetm; teeth that are inducing irregularities in the mouths of the young, particularly when they exhibit symptoms of a rapid and overmastering decay, are such as will need the skilful application of the numerous well-made forceps, and even take the power of diagnosing when and what teeth should be retained or extracted out of the category of empirical rules.

Frequently, however, we are not consulted, and a person will rush into our operating-room and demand the immediate extraction of a fairly good organ simply because it pains. Likely as not they will pitch upon the wrong tooth. and a contest arises between the patient and his dentist as to what is right to be done, and in this, as everything else, it will be found that the stronger will prevails, for, to use the words of a patient for whom I declined to remove a sound tooth that was condemed by its owner, "The person who feels the pain is the best judge as to which tooth to refer it."

Undoubtedly there are many cases where the pain is so intense that to the mind of the sufferer the only relief is the extraction of the tooth, but in the present time, with the power in our hands of devitalizing the pulp, in a large majority of cases, perhaps in all but under exceptional circumstances, more permanent and immediate relief can be given than by extraction. For assuming the worse condition of local aggravation, the pulp being destroyed, the surrounding tissues become rapidly amenable to treatment. The tooth, ceasing to pain, can readily have the pulp-cavity and roots emptied of the remaining slough, the chamber and canals being permanently plugged, and the useless walls reduced by excision to a level with the surrounding tissues.

The treatment of the anterior or single-rooted teeth, in consequence of their importance in restoring the natural appearance so perfectly, through the means of pivoted crowns, has long been under successful control.

The excision of one of these teeth when taken above the pulp chamber is comparatively a painless operation, in many cases the entire pulp coming away with the amputated portion; the root on being plugged either with the pivot of the newly adapted crown, or by means of any water-tight plug, becomes a restoration of the parts more natural and more permanently normal than can be produced by extraction under the most favourable circumstances.

That which has been done so frequently and so successfully for the teeth with a single pulp canal is also capable of being done with teeth of a larger number of both roots and canals. Undoubtedly the molars are larger and stronger organs, and the excision of their crown, if tolerably firm, might require a greater hand power than every dentist possesses; for the slightest deviation from rigid firmness and steadiness of hand is liable to dislocate the tooth in its socket, to give intense pain, and an after irritatian that not only may require a prolonged treatment, but probably vitiate the success of the operation altogether.

The compound character of the pulps of the posterior teeth is another source of difficulty, for it is scarcely probable that the excising power shall be so equally distributed that the branches of the pulp which traverse the different roots shall be simultaneously severed; consequently the force that ruptures the pulp of the anterior root of a molar tooth may only stretch that of the posterior, which, being done for the smallest amount of calculable time, must induce exquisite pain.

It appears to me, therefore, that the devitalization of the pulp previously to the removable of the crown is a thing to be desired, and the excision should be by a series of cuts, rather than by a single operation.

The roots of the excised tooth being clean and healthy, the pulp chamber and canals being carefully and hermetically sealed, the alveolar processes of the jaws are preserved and the mouth is retained in a condition more in accordance with its natural appearance, and less liable to vary for a long series of years than when the teeth are entirely removed.

I am aware that many practitioners advocate this mode of practice as conscientiously as their patients will admit of of their doing; but I think that it would largely advance the power of their advice and give increased confidence to their treatment if the subject received a full discussion by the profession, and it went forth as the dictum of this society, that the roots of teeth retained in a healthy condition is a thing to be desired, and that the preservation of the alveolar walls is synonymous with a youthful and healthy expression of the face.—*The Dental Record.*

